# Identification of a Recombinant Human Interleukin-12 (rhIL-12) Fragment in Non-Reduced SDS-PAGE

**DOI:** 10.3390/molecules24071210

**Published:** 2019-03-28

**Authors:** Lei Yu, Yonghong Li, Lei Tao, Chuncui Jia, Wenrong Yao, Chunming Rao, Junzhi Wang

**Affiliations:** National Institutes for Food and Drug Control, Beijing 100050, China; yulei@nifdc.org.cn (L.Y.); liyh@nifdc.org.cn (Y.L.); taolei01@nifdc.org.cn (L.T.); chuncui319@163.com (C.J.); yz1322@126.com (W.Y.)

**Keywords:** rhIL-12, purity, SDS-PAGE, SEC-HPLC, fragment, non-covalent binding

## Abstract

During the past two decades, recombinant human interleukin-12 (rhIL-12) has emerged as one of the most potent cytokines in mediating antitumor activity in a variety of preclinical models and clinical studies. Purity is a critical quality attribute (CQA) in the quality control system of rhIL-12. In our study, rhIL-12 bulks from manufacturer B showed a different pattern in non-reduced SDS-PAGE compared with size-exclusion chromatography (SEC)-HPLC. A small fragment was only detected in non-reduced SDS-PAGE but not in SEC-HPLC. The results of UPLC/MS and N-terminal sequencing confirmed that the small fragment was a 261–306 amino acid sequence of a p40 subunit of IL-12. The cleavage occurs between Lys260 and Arg261, a basic rich region. With the presence of 0.2% SDS, the small fragment appeared in both native PAGE and in SEC-HPLC, suggesting that it is bound to the remaining part of the IL-12 non-covalently, and is dissociated in a denatured environment. The results of a bioassay showed that the fractured rhIL-12 proteins had deficient biological activity. These findings provide an important reference for the quality control of the production process and the final products of rhIL-12.

## 1. Introduction

Interleukin-12 (IL-12) is a key inflammatory cytokine that critically influences Th1/Tc1-T cell responses at the time of an initial antigen encounter [1,2]. A growing number of studies have shed light on its potential in cancer immunotherapy [3,4]. During the past two decades, IL-12 has emerged as one of the most potent cytokines in mediating antitumor activity in a variety of preclinical models and clinical studies [5,6,7]. IL-12 has multiple biological functions, though most importantly, it bridges early nonspecific innate resistance and the subsequent antigen-specific adaptive immunity. A remarkable function of IL-12 is its ability to induce interferon γ (IFNγ) release from natural killer (NK) cells as well as CD4^+^ and CD8^+^ T cells [8,9]. Increasingly, pharmaceutical companies have invested in the research and development of recombinant human IL-12 (rhIL-12) [5,10,11]. One such company in China has obtained a national first class clinical approval for rhIL-12 injection. Aside from this, some IL-12 fusion proteins were developed to minimize adverse effects, such as huBC1-IL-12, F8-IL-12 and IL-12-SS1 (Fv), as well as IL-12 gene therapy products [8,11,12,13].

Native IL-12 is a heterodimer formed by two subunits, p40 (306 amino acids) and p35 (197 amino acids), that are bridged by an inter-subunit disulfide bond between Cys177 of p40 and Cys74 of p35, with three potential *N*-glycosylation sites [14,15]. Genes of the subunits p40 and p35 were located in different chromosomes in the human genome. The common preparation strategy for rhIL-12 is that *p40* and *p35* cDNA sequences were inserted into either two different vectors or one vector with two different promoters, and then transfected into a mammalian expression system [5,10]. As a recombinant protein drug, purity is a critical quality attribute (CQA) in the quality control system of rhIL-12. A purity test is essential for lot release and stability testing. The conventional analytical methods include chromatography and electrophoresis methods, such as size-exclusion chromatography (SEC), ion-exchange chromatography (IEC), denaturing protein gel electrophoresis (SDS-PAGE), capillary electrophoresis (CE)-SDS and capillary isoelectric focusing (cIEF) [16]. Aside from this, a liquid chromatography-mass spectrometry (LC-MS)-based multi-attribute method (MAM) has recently become a research hotspot [17,18]. As stated in the ICH Q6B, ‘the determination of absolute, as well as relative purity, presents considerable analytical challenges, and the results are highly method-development.’ Therefore, the purity must be assessed by a combination of analytical procedures. For most recombinant protein drugs, a combination of SDS-PAGE and SEC-HPLC are recommended. In our study, the purity of rhIL-12 bulks from manufacturer B was determined by non-reduced SDS-PAGE and SEC-HPLC, but the results were inconsistent. A small fragment was detected in non-reduced SDS-PAGE but not in SEC-HPLC. We used UPLC/MS and N-terminal sequencing to identify the fragment and then attempted to find out the cause of the cleavage and its effect on biological activity.

## 2. Results and Discussion

### 2.1. Purity Determination of rhIL-12 Samples by Non-Reduced SDS-PAGE and SEC-HPLC

**Figure 1:**
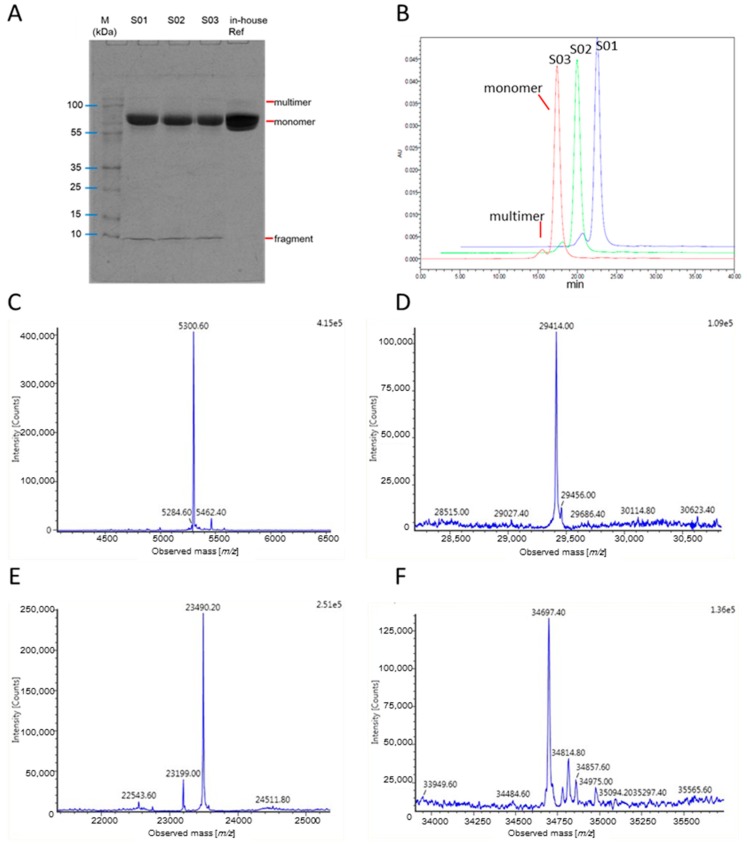
Purity and molecular weight determination of rhIL-12. (**A**,**B**) Purity determination of rhIL-12 bulks (S01, S02 and S03) by non-reduced SDS-PAGE and SEC-HPLC. (**C**,**D**) Molecular weight of p40 fragments by MS for rhIL-12 sample S01. (**E**) Molecular weight of p35 by MS for rhIL-12 sample S01. (**F**) Molecular weight of intact p40 by MS for rhIL-12 in-house reference.

Three batches of rhIL-12 bulks (S01, S02 and S03) from manufacturer B were tested by non-reduced SDS-PAGE and SEC-HPLC. The electrophoretogram and chromatogram are shown in Figure 1A,B, and the relative percentage contents are listed in Table 1. High molecular proteins, generally known as protein multimers, were detected in both assays, but the relative percentage contents in SEC-HPLC were significantly higher than those in SDS-PAGE, which may be caused by the different running system—native for SEC-HPLC and denatured for SDS-PAGE. The denaturation led most of the non-covalent multimers to be depolymerized, so the multimers in SDS-PAGE were generally significantly lower than in SEC-HPLC. However, fragments were only detected in non-reduced SDS-PAGE, and the relative percentage contents exceeded 7%. It was necessary to figure out the component of the small fragment present in non-reduced SDS-PAGE but absent in SEC-HPLC, as well as its origin and whether it was produced during the production process or during the testing process. No obvious small fragment was found in non-reduced SDS-PAGE for an rhIL-12 in-house reference (Figure 1A), suggesting that the fragment was not produced during SDS-PAGE. All test samples were bulks without any ingredients (such as a stabilizer) and were stored at −70 °C since prepared, which was conducted for over two years for the in-house reference and for a few months for the S01, S02 and S03 batches. It is generally recognized that proteins should be stable at −70 °C for an extended period of time (ultimately for a period of years). The in-house reference in this case had been stored for an even longer period, suggesting that the small fragment was not produced during storage. Thus it could be a product-related impurity or a process-related impurity originally existing in the rhIL-12 bulks. As an unknown protein impurity, it may bring about safety risks (such as toxicity or immunogenicity). We tried to identify the small fragment in SDS-PAGE in the following study.

### 2.2. Identification of rhIL-12 Fragment by UPLC/MS and N-Terminal Sequencing

UPLC/MS was employed to detect if there was any cleavage in the rhIL-12 peptides. For most glycoproteins, N-linked sugar chains are complex and heterogeneous, and are always removed for the determination of molecular weight (MW) by MS. The rhIL-12 samples were denatured by dithiothreitol (DTT) and deglycosylated by PNGase F before analysis. MW results are listed in Table 2. For subunit p35, although *O*-glycosylation caused heterogeneity in its MW, the measured and theoretical MWs were basically matched (Figure 1E). As for subunit p40, it is composed of 306 amino acids and its theoretical MW is 34698.03 Da. The measured value of the rhIL-12 in-house reference was 34697.40 Da (Figure 1F), which is highly consistent with the theoretical value. However, no intact p40 subunit was found in the rhIL-12 sample from manufacturer B. Instead, two fragments of 5.3 kDa and 29.4 kDa were detected (Figure 1C,D). The 29.4 kDa and 5.3 kDa fragments were consistent with the 1–260 amino acid sequence and the 261–306 amino acid sequence of subunit p40, respectively, which suggests that a cleavage did occur in subunit p40, and that the cleavage site was between Lys260 and Arg261, a dibasic site, as listed in Table 2. Since the inter-subunit disulfide bond is between the Cys177 of subunit p40 and the Cys74 of subunit p35 [14], the 29.4 kDa fragment should still be linked to subunit p35 covalently.

To verify whether the small peptide in the non-reduced SDS-PAGE was the 5.3 kDa fragment of subunit p40, we further identified it by N-terminal sequencing. The measured sequence of 16 N-terminal amino acids was REKKDRVFTDKTSATV, which was completely consistent with the theoretical N-terminal sequence of the 5.3 kDa fragment, confirming that the small peptide in non-reduced SDS-PAGE was the 5.3 kDa fragment of subunit p40. The Cycles 2 to 6 are shown in Figure S1. The reason why it was only present in non-reduced SDS-PAGE but absent in SEC-HPLC may be due to the different running systems of SEC-HPLC (native) compared with SDS-PAGE (denatured). The fragment may bind to the remaining part of IL-12 non-covalently in a native environment but dissociate in a denatured environment. To test this further, we next evaluated the effect of the denaturant.

### 2.3. Effect of Denaturant on rhIL-12 Pattern in Native PAGE and SEC-HPLC

**Figure 2:**
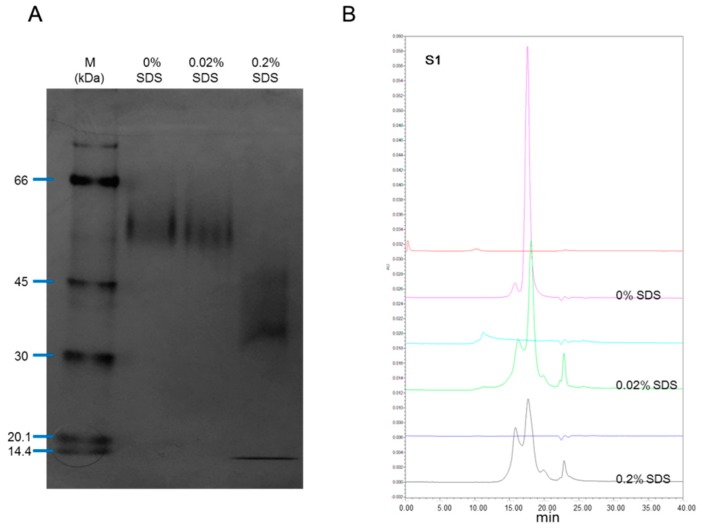
Effect of SDS on rhIL-12 pattern in native PAGE and SEC-HPLC. The rhIL-12 bulk S01 was treated by 0%, 0.02% and 0.2% SDS before analysis. (**A**) Native PAGE. (**B**) SEC-HPLC.

The rhIL-12 sample S01 was treated with 0%, 0.2% or 0.02% SDS for 10 min at room temperature before being tested by native PAGE and SEC-HPLC. In non-denatured PAGE, no fragment was found without the presence of SDS or with the presence of 0.02% SDS, but in the case where 0.2% SDS was employed, the small fragment reappeared (Figure 2A). In SEC-HPLC, with the presence of SDS (both 0.02% and 0.2%), the small fragment appeared (Figure 2B). SDS is amphipathic in nature, which allows it to unfold both polar and nonpolar sections of a protein structure. In SDS concentrations above 0.1 mM (0.003%), the unfolding of proteins begins, and above 1 mM (0.03%), most proteins are denatured [19]. These results suggest that the 5.3 kDa fragment bound to the remaining part of IL-12 non-covalently in a non-denatured environment, and dissociated with the presence of SDS. This explains why the 5.3 kDa fragment was only visible in SDS-PAGE but not in SEC-HPLC. 

As for the inconsistency between native PAGE and SEC-HPLC with the presence of 0.02% SDS, this may be caused by a different reaction time. For PAGE, all samples were loaded onto the gel at the same time, but for SEC-HPLC, samples were analyzed in sequence (0%, 0.02% and 0.2% SDS), meaning that the reaction time of samples for SEC-HPLC were unequal and longer than for PAGE. The longer reaction time of SDS always brings about greater efficiency in terms of denaturation.

### 2.4. Cleavage Site in 3D Structure of rhIL-12

**Figure 3:**
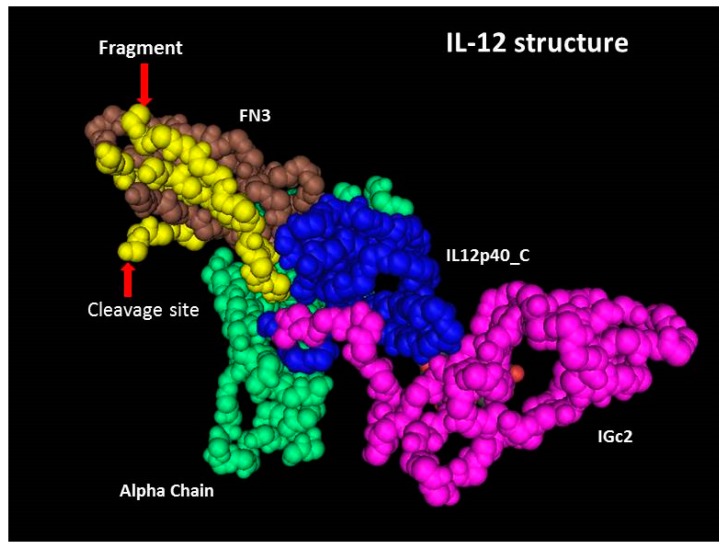
3D structure of IL-12. The alpha chain (green) is subunit p35. Subunit p40 consists of three domains: FN3 (brown), rhIL-12p40_C (blue) and IGc2 (pink). The yellow region is the 5.3 kDa fragment, a part of the FN3 domain. This 3D structure was obtained from the Protein Data Bank (PDB) website (ID: 1F45).

The p40 subunit consists of three domains, FN3, rhIL-12p40_C and IGc2 [20]. As shown in Figure 3, the cleavage occurs between Lys260 and Arg261, and a 5.3 kDa fragment is located in the FN3 domain of subunit p40, which is tightly folded. The cleavage site is a basic rich sequence (Lys-Ser-Lys-Arg-Glu-Lys-Lys) exposed on the surface of the molecule. Lys260-Arg261 is a dibasic site, liable to be targeted by endogenous protease [21,22,23], and residual proteases used for the removal of protein purification tag(s) (if any) may have a nonspecific effect on the site. Other than this, low pH conditions could also induce the instability of basic amino acids. The cleavage may occur during the production or the purification process. Intermediate products at different steps of the process should be analyzed to figure out at which step the cleavage occurred and to develop an appropriate strategy to avoid this occurrence.

In the spatial structure of IL-12, the 5.3 kDa sequence is located in the C-terminal of subunit p40, and binds tightly with the rest of the FN3 domain, which should be the reason for its absence in native PAGE and SEC-HPLC. However, in non-reduced SDS-PAGE, a denatured environment destroyed the non-covalent bond and the fragment was disassociated and finally appeared in the electrophoretogram. For proteins, non-reduced SDS-PAGE is usually the first choice as an assay of purity, not only because of its reliability and ease, but also because of its ability to separate the fragments binding to the principal component non-covalently. 

### 2.5. Influence of Cleavage on Bioactivity

**Figure 4:**
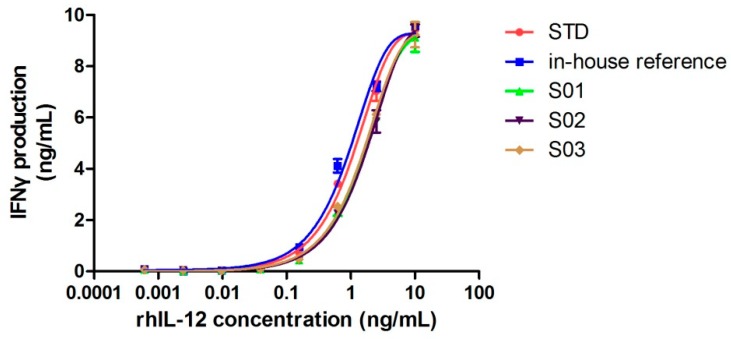
Dose-response curves of WHO biological standard for rhIL-12, the rhIL-12 in-house reference and rhIL-12 samples (S01–S03). Each plot represents the mean of two replicates.

Although cleavage occurred, if the spatial structure remained intact, proteins could still function properly. Next, we confirmed whether this cleavage had any negative influence on its bioactivity by comparing its specific activity with the rhIL-12 in-house reference (intact IL-12). The biological activity of rhIL-12 was determined by NK92MI/interferon γ release assay, which served to test its induction of interferon γ release in NK92MI cells. Figure 4 shows the dose-response curves of the World Health Organization (WHO) biological standard for rhIL-12, the rhIL-12 in-house reference and samples (S01–S03). The results of the protein content and biological activity are listed in Table 3. Three batches of fractured rhIL-12 (S01, S02 and S03) showed half the specific activity of intact rhIL-12. 

Although no intact IL-12 molecule was found in the rhIL-12 bulks from manufacturer B by MS, 50% of the total activities were reserved. The reason for this may be that half of the 5.3 kDa fragments were folded properly with the remaining part of the IL-12, forming an intact spatial structure, or that the incomplete spatial structure still retained partial activity, which should be further studied by spatial structure analysis. Nonetheless, the cleavage had a negative effect on the biological activity of rhIL-12. 

## 3. Materials and Methods

### 3.1. Materials

The WHO biological standard for IL-12 was obtained from the National Institute for Biological Standards and Control (NIBSC code: 95/544). The rhIL-12 in-house reference (bulk from manufacturer A, Qingdao, China) and samples S01, S02 and S03 (different batches of bulks from manufacturer B, Guangzhou, China) were archived samples that had been preserved at −70 °C in our laboratory. 

### 3.2. Electrophoresis Analysis

Purity was evaluated by non-reduced SDS-PAGE performed on a 4–20% SDS-tris-glycine gel (Thermo Fisher Scientific, Carlsbad, CA, USA). Samples were diluted in a non-reducing SDS sample buffer and heated at 95 ± 5 °C for 5 min with 10 μg of each sample loaded onto the gel. The samples were separated by electrophoresis and the gel was stained with 0.25% w/v Coomassie R-250 (Bio-Rad, Hercules, CA, USA), destained for clarity, and scanned. The relative percentage contents were calculated using the area normalization method. For native PAGE, SDS was excluded from the electrophoresis system. 

### 3.3. Size-Exclusion Chromatography Analysis

LC separation was performed on a Waters2695 system with a TSK-GEL G3000 SWXL column (300 mm × 7.8 mm, Tosoh, Japan). The injection volume was 50 μL. The flow rate was 0.5 mL/min using an elution buffer of 40 mM phosphate buffer containing 300 mM sodium sulfate (pH 7.2) and the column temperature was maintained at 25 °C. The detection was performed on a Waters2489 UV detector (Waters, Milford, MA, USA) at 280 nm. Data were acquired and processed using Waters Empower (Waters Corporation, Milford, MA, USA). The relative percentage contents were calculated by the area normalization method. 

### 3.4. UPLC/MS

The rhIL-12 samples were denatured by 10mM DTT (Sigma, St. Louis, MO, USA) and deglycosylated by PNGase F (New England Biolabs, Beijing, China), then analyzed by the Acquity UPLC system connected online to a Xevo G2-S mass spectrometer (Waters Corporation, Milford, MA, USA). The column was a Waters BEH300 C4 column (2.1 mm × 50 mm, 1.7 μm particle). The flow rate was 0.2 mL/min using a gradient from 5% to 50% Solvent B (Solvent B being 0.1% formic acid in acetonitrile, Solvent A being 0.1% formic acid in water) in 7 min at a column temperature of 35 °C. The scan range of the mass spectrometric was *m*/*z* 500–3000. Data were acquired and processed using UNIFI 1.6 (Waters Corporation, Milford, MA, USA). 

### 3.5. N-Terminal Sequencing

The rhIL-12 sample (S01) was condensed to about 2 mg/mL by ultrafiltration using 3 kDa centrifugal filters (Merck Millipore Ltd., Tullagreen, Ireland). Then, 32 μL of the sample (64 μg) was mixed with 8 μL non-reducing SDS sample buffer (5×) and heated at 95 ± 5 °C for 5 min, and subsequently loaded onto a 4–20% Tris-glycine gel and separated by SDS-PAGE. Proteins were transferred electrophoretically onto a polyvinylidene fluoride (PVDF) membrane. The small fragment was excised and subjected to 16 cycles of N-terminal sequence analysis using a PPSQ-53A protein sequencer (Shimadzu, Kyoto, Japan).

### 3.6. Measurement of rhIL-12 Concentration and Bioactivity

The protein contents of the rhIL-12 in-house reference and samples were determined by a Pierce™ BCA protein assay kit (Thermo scientific, Rockford, IL, USA) according to the manufacturer’s instructions. The bioactivity of rhIL-12 was determined by quantifying IFN-γ secretion by the IL-12-responsive NK-92MI cell line (American Type Culture Collection, Manassas, VA, USA) cultured in complete media consisting of Minimum Essential Medium α (MEMα) supplemented with 12% fetal bovine serum (FBS), 12% horse serum, 1% penicillin/streptomycin, 0.2 mM inositol, 0.02 mM folic acid and 0.1 mM 2-mercaptoethanol. In brief, cultured NK-92MI cells were seeded in a 96-well plate at 20,000 cells/well. The WHO biological standard for IL-12, the rhIL-12 in-house reference and the samples were added to the cells at final concentrations of 10 ng/mL~0.0006 ng/mL. IFN-γ concentrations in NK92-MI supernatants after 24 hours were quantified using an IFN-γ ELISA kit (BD Biosciences, Franklin Lakes, NJ, USA) according to the manufacturer’s instructions.

## 4. Conclusions

Collectively, all our results proved that the small fragment in non-reduced SDS-PAGE was a 261–306 amino acid sequence of subunit p40, located in the FN3 domain, a tightly folded domain in the IL-12 spatial structure. The fragment could bind to the remaining part of IL-12 non-covalently and dissociate in a denatured environment. The cleavage site was found to be a basic rich sequence exposed on the surface of the molecule. The cleavage may occur during the production or the purification processes. Fractured rhIL-12 proteins had deficient biological activity. Additionally, the cleavage was not unique, found not only in samples from manufacturer B but also in samples from another manufacturer, manufacturer C (data not shown). Thus, it is necessary to find the cause of the cleavage and develop an appropriate strategy to avoid its occurrence. Our work provides an important reference for the quality control of the production process and final products of rhIL-12, as well as an improvement in the production technology. This study reveals the importance of purity determination through a combination of analytical procedures with different principles. 

## Figures and Tables

**Table 1:** Purity of recombinant human interleukin-12 (rhIL-12) bulks by SDS-PAGE and size-exclusion chromatography (SEC)-HPLC.

Sample ID	Multimer (%) ^a^		Monomer (%) ^a^		Fragment (%) ^a^	
	SDS-PAGE	SEC-HPLC	SDS-PAGE	SEC-HPLC	SDS-PAGE	SEC-HPLC
S01	0.69	4.19	92.00	95.81	7.31	–^b^
S02	0.71	2.96	91.32	97.04	7.97	–^b^
S03	0.98	4.60	91.29	95.40	7.73	–^b^

^a^. The relative percentage contents were calculated by the area normalization method. ^b^. Not detected.

**Table 2:** Molecular weight (MW) of rhIL-12 (S01) by MS.

Subunit	Amino Acid Sequence	Theoretical MW (Da)	Measured MW (Da)	Error (Da)	Relative Error (ppm)
p40	1–260	29415.12 ^a^	29414.00	1.12	38
	261–306	5300.93	5300.60	0.33	62
p35	1–197	22544.21 ^b^	22543.60	0.61	27
		23200.80 ^c^	23199.00	1.80	78
		23492.06 ^d^	23490.20	1.86	79

^a^. One *N*-glycosylation (Glu→Gln) caused an increase of 0.98 Da; ^b^. Two *N*-glycosylations (Glu→Gln) caused an increase of 1.96 Da; ^c^. *O*-glycosylation (GlcNAc-Man-SA) caused an increase of 656.59 Da based on b; ^d^. *O*-glycosylation (GlcNAc-Man-2SA) caused an increase of 947.85 Da based on b.

**Table 3:** The results of protein content, biological activity and specific activity.

Samples	Protein Content (mg/mL, Mean of Three Replicates)	Biological Activity (units/mL, Mean of Three Replicates)	Specific Activity (units/mg)
In-house reference	1.78	1.57 × 10^7^	8.81 × 10^6^
S01	0.35	1.56 × 10^6^	4.45 × 10^6^
S02	0.34	1.46 × 10^6^	4.31 × 10^6^
S03	0.35	1.57 × 10^6^	4.48 × 10^6^

## Supplementary Materials

The following are available online, Figure S1: Determination of N-terminal amino acid sequence by Edman degradation.
