# Supplementary figures and images for: Identification of a Recombinant Human Interleukin-12 (rhIL-12) Fragment in Non-Reduced SDS-PAGE
Source: Molecules. 2019 Mar 28;24(7):1210. doi: 10.3390/molecules24071210 (PMC6479496; doi:10.3390/molecules24071210)

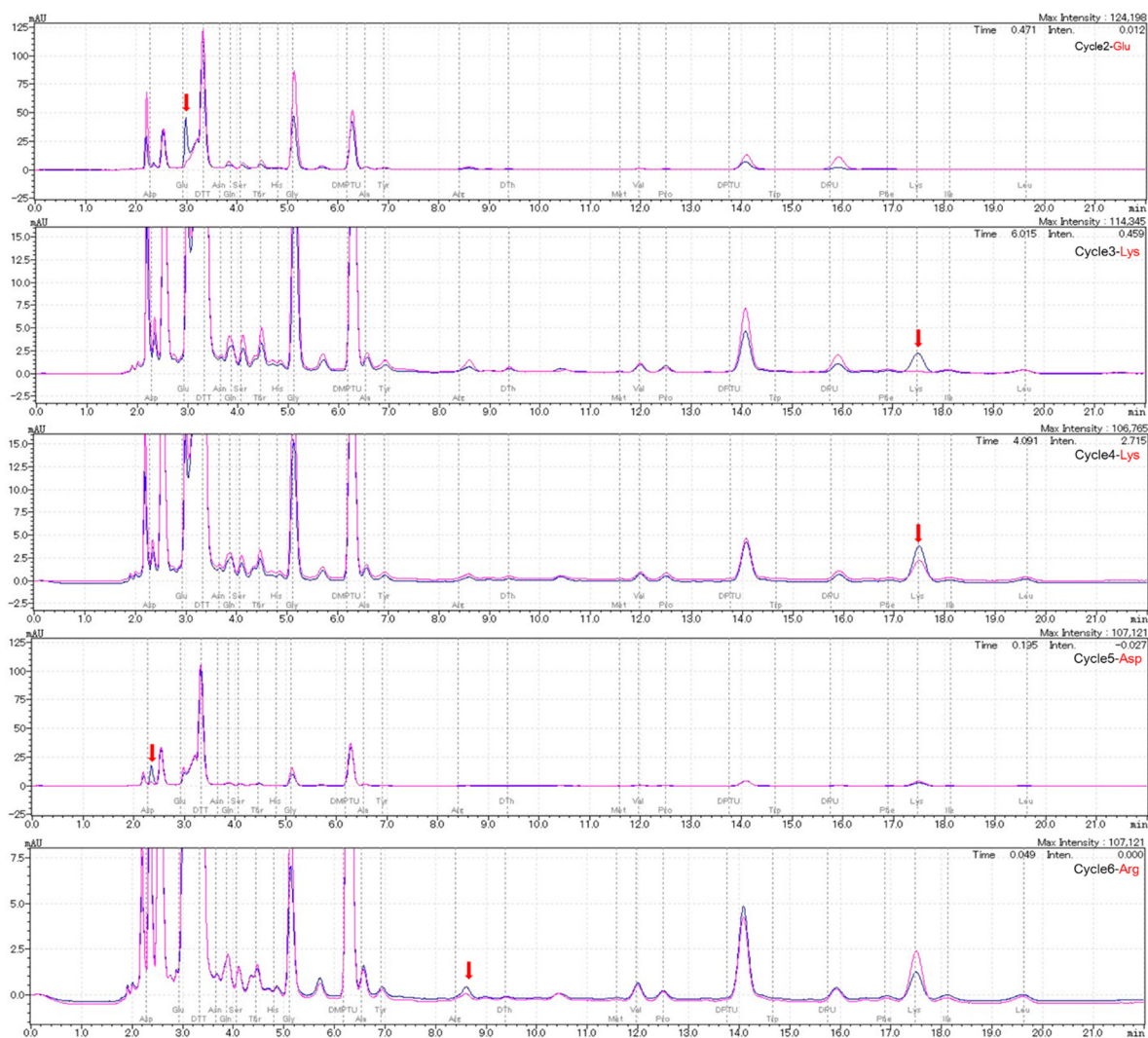

Supplement: Supplementary file 1 [file molecules-24-01210-s001.pdf]
